# Supplementary material for: Two Types of Discontinuous Percolation Transitions in Cluster Merging Processes
Source: Sci Rep. 2015 Jul 7;5:11905. doi: 10.1038/srep11905 (PMC5387406; doi:10.1038/srep11905)
Supplement: Supplementary Information [file srep11905-s1.pdf]

Supplementary Information

Two Types of Discontinuous Percolation Transitions in Cluster Merging Processes

Y. S. Cho[1] and B. Kahng[1, *]

[1]Department of Physics and Astronomy, Seoul National University, 151-747, Korea

*bkahng@snu.ac.kr

Here we present supplementary information that accompanies the manuscript entitled "*Two types of discontinuous percolation transitions in Cluster Merging Processes.*"

I. Numerical testing of the necessary condition for various models

We check the necessary conditions for various models. Detailed explanations on whether the necessary conditions are fulfilled or not are presented in the caption of each figure.

A. For models showing type-II discontinuous percolation transitions

A-1) The solvable TCA model introduced in the main text (Fig.S1)

A-2) The BFW model in Ref. [24] (Fig.S2)

A-3) The percolation model in a hierarchical lattice with long-range connections [26] (Fig.S3)

A-4) The model with the half-restricted process [25] (Fig.S4)

B. For a model showing a type-I discontinuous percolation transition

B-1) The Gaussian model [20] (Fig.S5)

C. For a model showing a continuous percolation transition

C-1) The explosive percolation model with product rule [4] (Fig.S6)

II. How the symmetry breaking dynamic rule leads to a type-II DPT.

We introduce the necessary conditions for different species of clusters and check how a type-II DPT is induced by the symmetry breaking rule in the following models.

D. The necessary conditions for the case with symmetry breaking rule

E. Numerical check of those conditions for several models showing type-II DPTs.

E-1) The TCA model introduced in the main text (Fig.S7)

E-2) The BFW model [24] (Fig. S8)

E-3) The half-restricted process model [25] (Fig. S9)

III. Symmetry breaking dynamic rule for type-II synchronization transition

I. Numerical testing of the necessary condition for various models

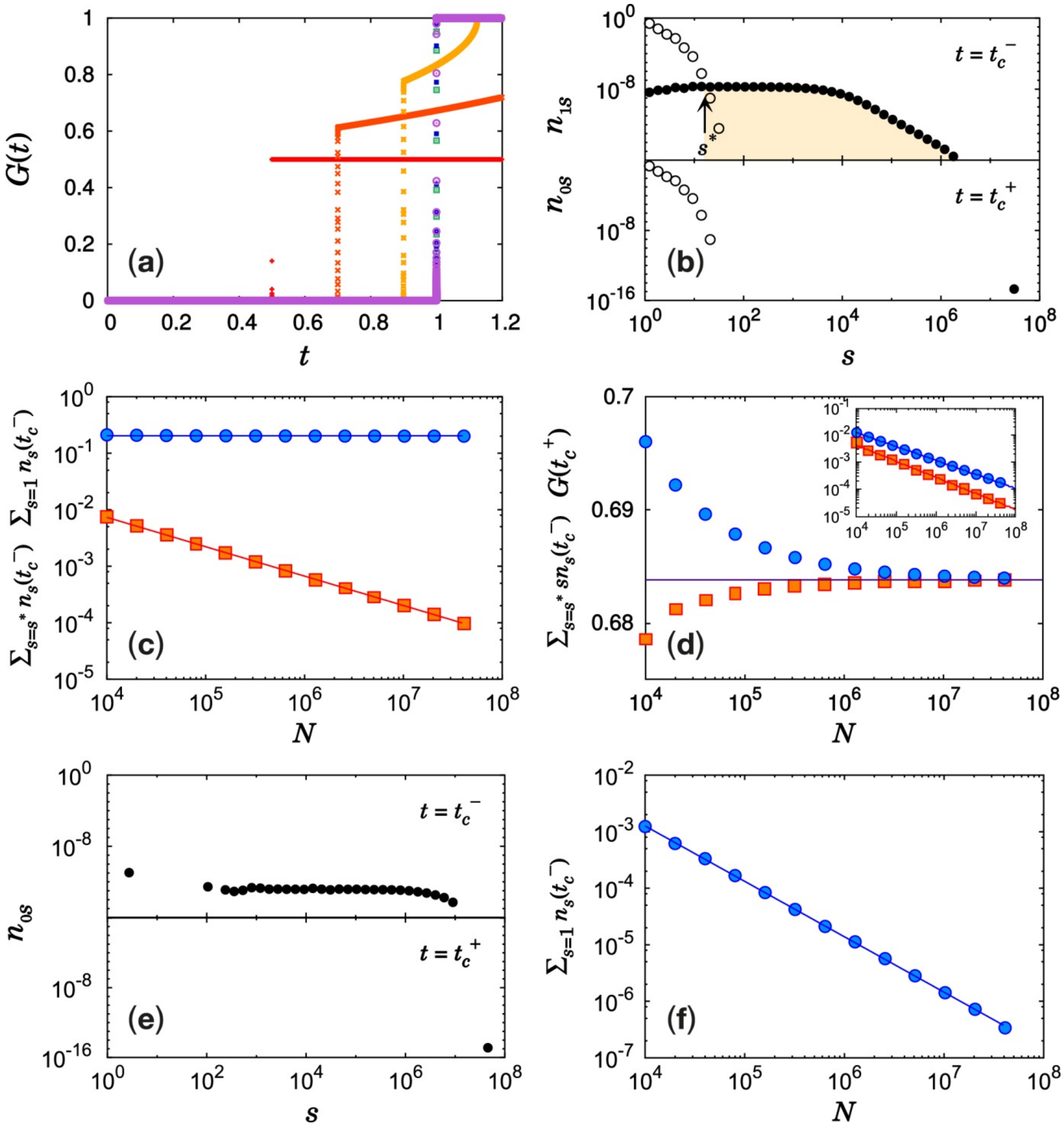

Fig.S1 A-1 Checking the necessary condition for the solvable TCA model

(a) Plot of $G(t)$ vs t for $p=0,\ 0.2,\ 0.4,\ 0.6,\ 0.8$ and 1.0 from left to right for a fixed system size $N=2^{12}\times 10^4$. As p is increased, t_c is delayed. $G(t)$ exhibits a type-I percolation transition at $t_c=1$ for $p=0.6,\ 0.8$ and 1.0. For $p=0$, PT is also of type I, in which single-species merging dynamics takes place. **(b)** Plot of the size distributions of black clusters n_{0s} (●) and white clusters n_{1s} (○) for $p=0.3$ and $N=2^{12}\times 10^4$. Large black clusters and small white clusters coexist at t_c^-. Here, s^* denotes the crossing

point of $n_{0s}(t_c^-)$ and $n_{1s}(t_c^-)$. As shown in the lower panel, all black clusters and some white clusters merge during $\Delta t = t_c^+ - t_c^-$, and consequently a large black cluster forms at t_c^+. **(c)** Plot of $\sum_{s=s^*}^{\infty} n_s(t_c^-)$ (□) and $\sum_{s=1}^{\infty} n_s(t_c^-)$ (○) vs N. The slopes of the guidelines are 0 for (○) and -0.52 (□). **(d)** Plot of $\sum_{s=s^*}^{\infty} s n_s(t_c^-)$ (□) and $G(t_c^+)$ (○) vs N. The two data sets converge to the value $y_0 \approx 0.68$. Inset: plot of $y_0 - \sum_{s=s^*}^{\infty} s n_s(t_c^-)$ (□) and $G(t_c^+) - y_0$ (○) vs N. The slopes of the guidelines are -0.52 and -0.60 from the top. The dataset for **(b-d)** were obtained for $p = 0.3$. **(e)** Plot of the size distribution of black clusters n_{0s} (●) for $p = 1.0$ (type-I DPT case) and $N = 2^{12} \times 10^4$. In this case, only black clusters are left at t_c^-. As shown in the lower panel, all the black clusters merge during $\Delta t = t_c^+ - t_c^-$, and consequently a large black cluster of size N forms at t_c^+. **(f)** We measure $\sum_{s=1}^{\infty} n_s(t_c^-)$ as a function of N for $p = 1.0$. $\sum_{s=1}^{\infty} n_s(t_c^-)$ decreases to 0 as $\sim N^{-0.98}$, which implies that the case of $p = 1.0$ satisfies the necessary condition for type-I DPT.

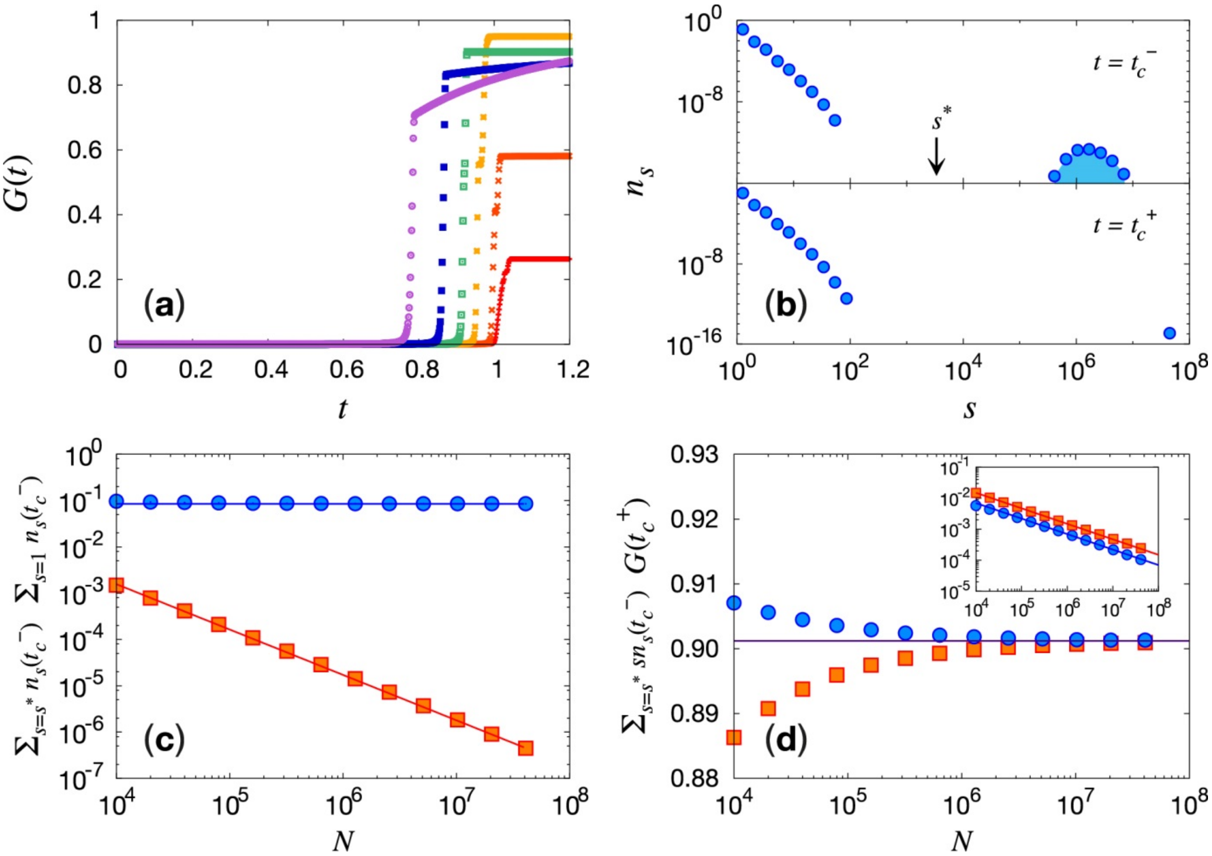

Fig.S2 A-2 Checking the necessary condition for the BFW model

(a) Plot of $G(t)$ vs t for $\alpha = 0.2,\ 0.4,\ 0.6, 0.7, 0.8$ and 0.9 from right to left for a system size of $N = 10^5$, where α is a control parameter given in the model. **(b)** Plot of $n_s(t)$ vs s for $\alpha = 0.7$ and $N = 2^{12} \times 10^4$. Large and small clusters coexist at t_c^- shown in the upper panel. s^* can be naturally chosen as a point between the regions of small and large clusters. As shown in the lower panel, all large clusters merge during the interval $\Delta t = t_c^+ - t_c^-$, and consequently form a large cluster. **(c)** $\sum_{s=1}^{\infty} n_s(t_c^-)$ (○) is independent of N and $\sum_{s=s^*}^{\infty} n_s(t_c^-)$ (□) decreases to 0 as the system size diverges. The exponent is about 0.98. **(d)** Plot of $G(t_c^+)$ (○) and $\sum_{s=s^*}^{\infty} sn_s(t_c^-)$ (□) seem to converge to the same value 0.90, indicated by the solid line. Inset: Plots of $G(t_c^+) - 0.90$ (○) and $0.90 - \sum_{s=s^*}^{\infty} sn_s(t_c^-)$ (□)vs N. Both quantities decay in a power-law manner with the exponent value -0.50 as shown in the Inset. The dataset for **(c)** and **(d)** were obtained for $\alpha = 0.7$.

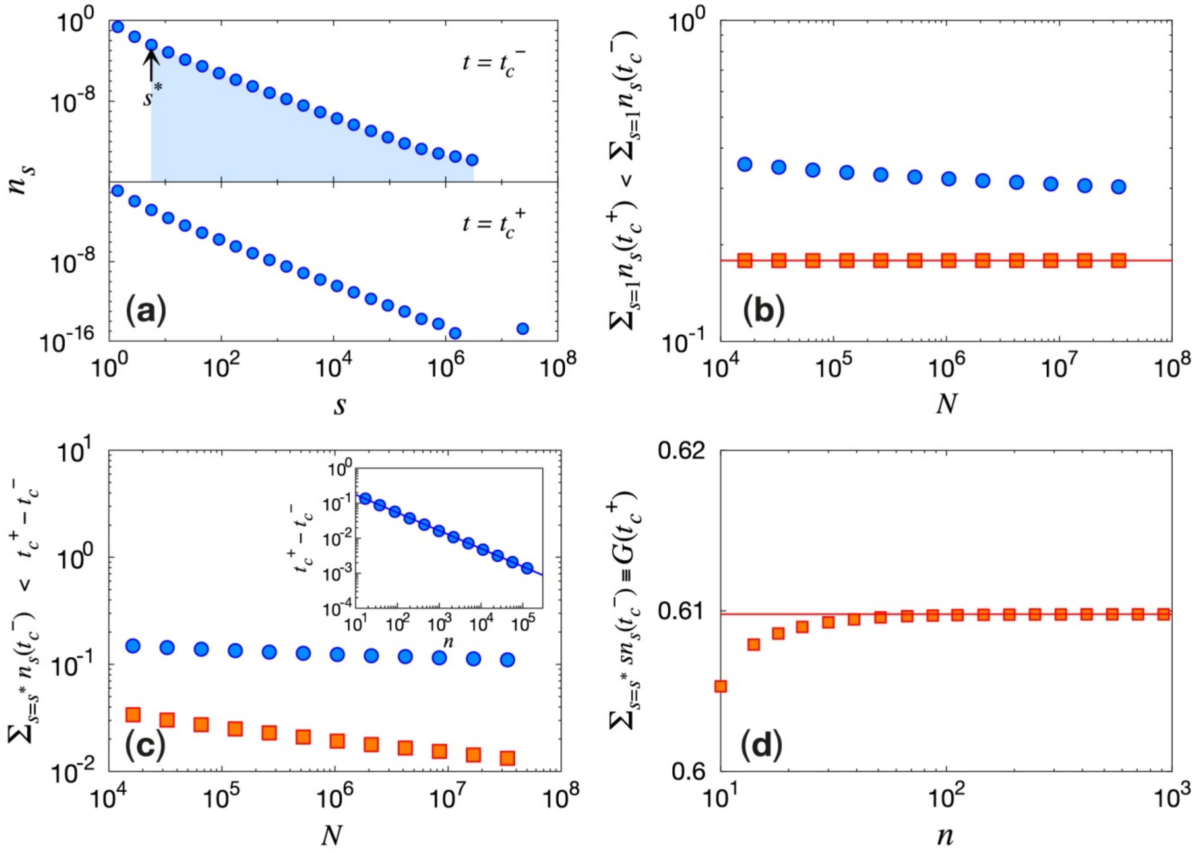

Fig.S3 A-3 Checking the necessary condition for the percolation in a hierarchical lattice with long-range connections

(a) Plots of $n_s(t_c^-)$ and $n_s(t_c^+)$ vs s for $N = 2^{25}$ in the upper and the lower panel, respectively. In this model, since $n_s(t_c^-)$ decays monotonically, we cannot determine s^* naturally as taken in the previous cases. However, we take s^* through the relation $\sum_{s=s^*}^{\infty} sn_s(t_c^-) \equiv G(t_c^+)$, where $t_c^+ = 1$. **(b)** We measure $\sum_{s=1}^{\infty} n_s(t_c^+)$ (□) and $\sum_{s=1}^{\infty} n_s(t_c^-)$ (○). We find that $\sum_{s=1}^{\infty} n_s(t_c^+)$ is independent of N, and thus $\lim_{N\to\infty} \sum_{s=1}^{\infty} n_s(t_c^-) \sim O(1)$ by the relation $\sum_{s=1}^{\infty} n_s(t_c^-) > \sum_{s=1}^{\infty} n_s(t_c^+)$. **(c)** We measure $\sum_{s=s^*}^{\infty} n_s(t_c^-)$ (□) and $t_c^+ - t_c^-$ (○). By the definition of s^*, $\sum_{s=s^*}^{\infty} n_s(t_c^-) < t_c^+ - t_c^- \to 0$ satisfies and thus, $\sum_{s=s^*}^{\infty} n_s(t_c^-) \to 0$. Inset: $t_c^+ - t_c^-$ decreases to 0 as $\sim n^{-0.51}$, where $n = \log_2 N$. **(d)** Finally, we measure the $\sum_{s=s^*}^{\infty} sn_s(t_c^-) \equiv G(t_c^+)$ as a function of n and find that $\sum_{s=s^*}^{\infty} sn_s(t_c^-)$ converges to 0.61, which is equivalent to the analytic result.

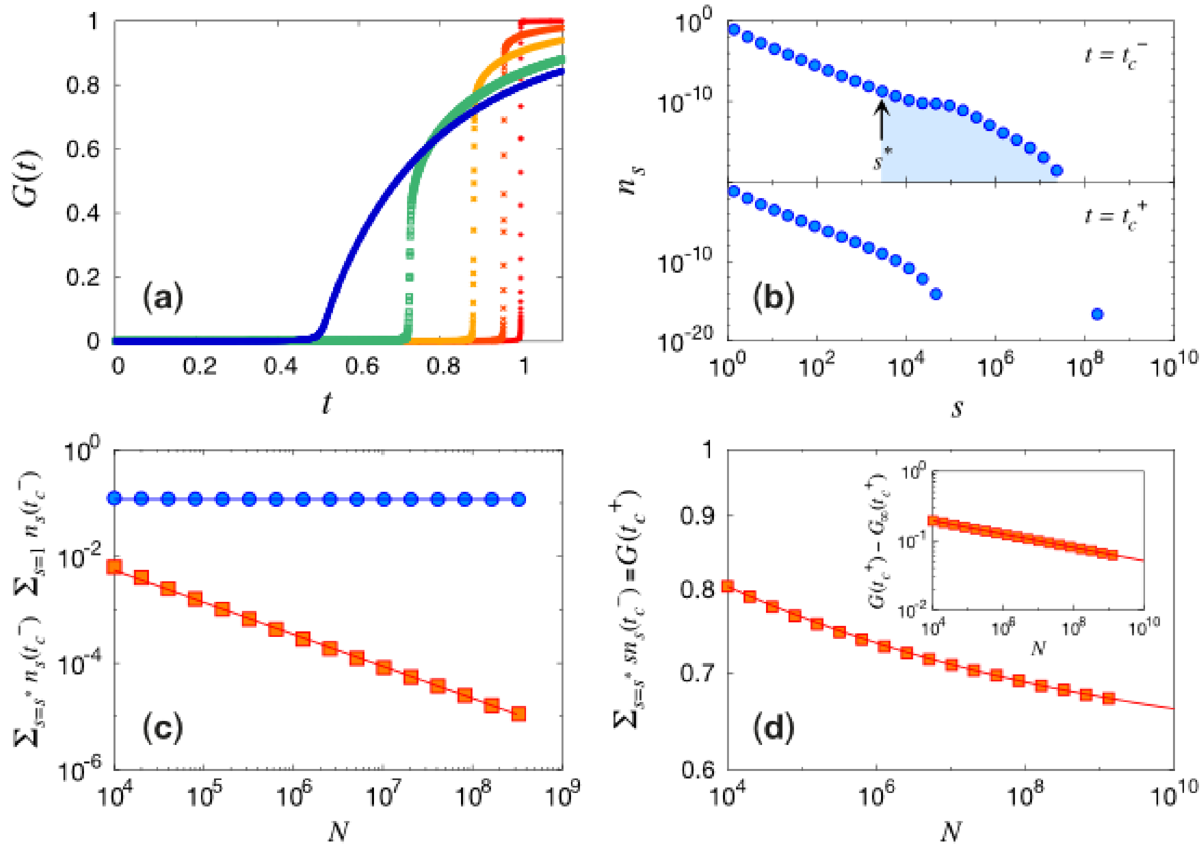

Fig.S4 A-4 Checking the necessary condition for a model with the half-restricted process

(a) Plot of $G(t)$ vs t for various values of $d = 0.2,\ 0.4,\ 0.6,\ 0.8$ and 1.0 for a system size of $N = 10^5$ from right to left, where d is a control parameter given in the model. **(b)** Plot of $n_s(t_c^-)$ vs s for $d = 0.5$ and $N = 2^{15} \times 10^4$ in the upper panel. Here, again s^* was determined using $\sum_{s=s^*}^{\infty} s n_s(t_c^-) \equiv G(t_c^+)$. After $\Delta t = t_c^+ - t_c^-$, a large cluster is formed as shown in the lower panel. **(c)** We plot $\sum_{s=1}^{\infty} n_s(t_c^-)$ (○) vs N to confirm its independency of N. We also confirm that $\sum_{s=s^*}^{\infty} n_s(t_c^-)$ (□) decreases to 0 as $\sim N^{-0.60}$. **(d)** $\sum_{s=s^*}^{\infty} s n_s(t_c^-) \equiv G_N(t_c^+)$ decreases with respect to N, but it seems to be saturated asymptotically. We estimated the saturated value by extrapolating the data to be $G_\infty(t_c^+) \approx 0.61$. Inset: It shows that $G_N(t_c^+) - G_\infty(t_c^+) \sim N^{-0.01}$. Numerical data for **(c)** and **(d)**. were obtained for $d = 0.5$.

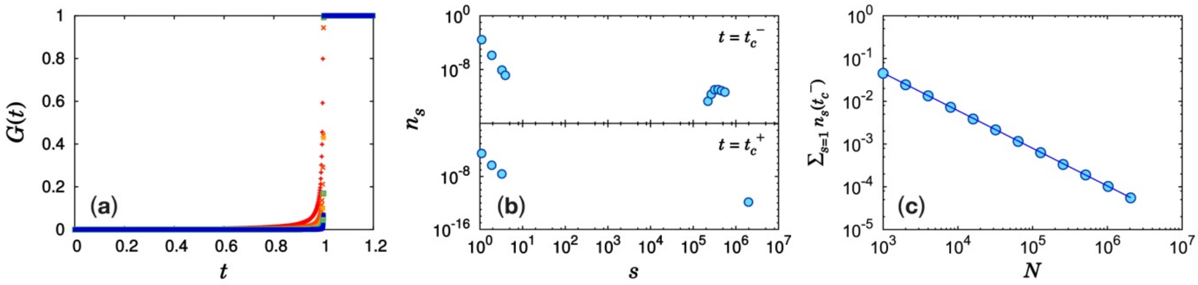

Fig. S5 B-1 Checking the necessary condition for the Gaussian model

(a) Plot of $G(t)$ vs t for $N = 10^5$ with parameter values $\alpha = 10^{-4}, 10^{-3}, 10^{-2}, 10^{-1}$ and 1 from the left, where α is a control parameter given in the model. **(b)** Plot of $n_s(t_c^-)$ vs s for $N = 2^{11} \times 10^3$ and $\alpha = 10^{-2}$ in the upper plot. Large and small clusters coexist at t_c^- as shown in the upper panel. As shown in the lower panel, all large clusters merge during the time interval $\Delta t = t_c^+ - t_c^-$, and consequently form a large cluster. **(c)** Plot of $\sum_{s=1}^{\infty} n_s(t_c^-)$ (○) vs N for $\alpha = 10^{-2}$. It decays in a power law manner as $N^{-0.88}$, which fulfills the necessary condition for type-I DPT.

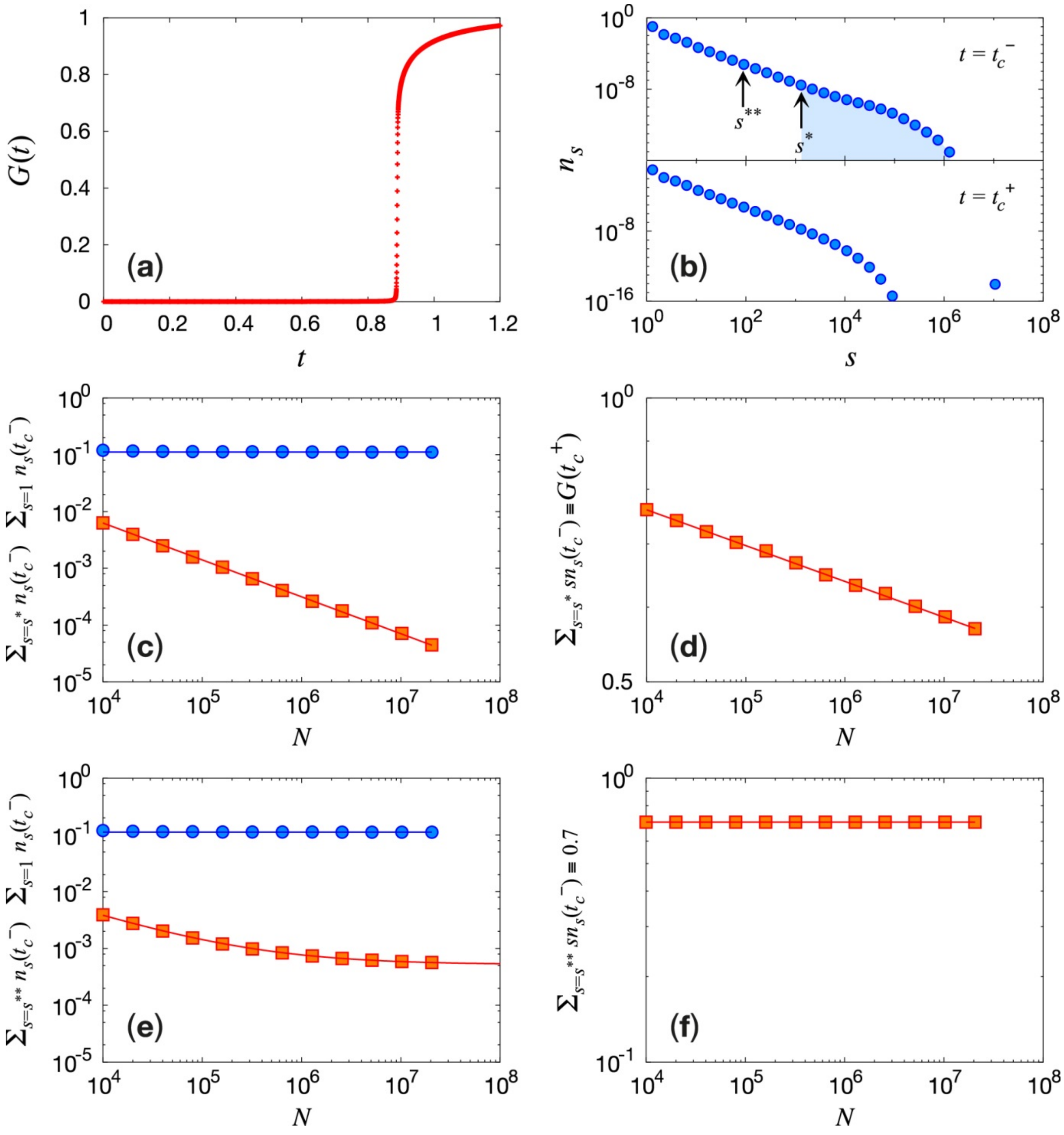

Fig. S6 C-1 Checking the necessary condition for the explosive percolation model with product rule

(a) Plot of $G(t)$ vs t for $N = 2^4 \times 10^4$. **(b)** Plot of $n_s(t_c^-)$ vs s for $N = 2^{12} \times 10^4$ in the upper panel. For this model, we were not able to determine s^* easily as the case of the TCA model. s^* and s^{**} are determined using $\sum_{s=s^*}^{\infty} sn_s(t_c^-) \equiv G(t_c^+)$ and taking $\sum_{s=s^{**}}^{\infty} sn_s(t_c^-) \equiv 0.7$, respectively. After a transient time interval $\Delta t = t_c^+ - t_c^-$, a large component is formed as shown in the lower plot. **(c)** $\sum_{s=1}^{\infty} n_s(t_c^-)$ (○) is

independent of system size and $\sum_{s=s^*}^{\infty} n_s(t_c^-)$ (□) decreases to 0 as $\sim N^{-0.71}$. **(d)** $\sum_{s=s^*}^{\infty} sn_s(t_c^-) \equiv G(t_c^+)$ decreases to 0 as $\sim N^{-0.05}$. **(e)** $\sum_{s=1}^{\infty} n_s(t_c^-)$ (○) is independent of system size and $\sum_{s=s^{**}}^{\infty} n_s(t_c^-)$ (□) decreases and approaches a certain finite value. **(f)** $\sum_{s=s^{**}}^{\infty} sn_s(t_c^-)$ is independent of N. As a result, this model does not fulfill both necessary conditions for type-I and type-II DPT and the type of the percolation transition becomes continuous in the thermodynamic limit.

II. How the symmetry breaking dynamic rule leads to a type-II DPT.

D. The necessary conditions for the case with symmetry breaking dynamic rule:

As clusters are merged, clusters are classified into two types 0 and 1 satisfying the following conditions.

(i) $\sum_{s=1}^{s*} n_{0s}(t_c^-) \to 0$ and $\sum_{s=s*}^{\infty} n_{0s}(t_c^-) \to 0$. These conditions lead to an abrupt PT, driven by merging clusters of type 0 themselves.

(ii) $\sum_{s=1}^{s*} sn_{0s}(t_c^-) \to 0$ and $\sum_{s=s*}^{\infty} sn_{0s}(t_c^-) = r\ (0 < r < 1)$. These conditions lead to a type-II DPT in which the order parameter is increased as much as r by merging clusters of type 0 themselves.

(iii) $\sum_{s=1}^{s*} n_{1s}(t_c^-) \sim O(1)$ and $\sum_{s=s*}^{\infty} n_{1s}(t_c^-) \to 0$. These conditions lead to a gradual increase of giant cluster by merging clusters of type 1 after the abrupt transition. Without these conditions, the giant cluster cannot grow continuously after a discontinuous percolation transition occurs.

(iv) $\sum_{s=1}^{s*} sn_{1s}(t_c^-) = 1 - r$. This is the complement to the condition (ii).

Combining the above necessary conditions, the following necessary conditions are obtained for a type-II DPT.

I-i) $\sum_{s=s*}^{\infty} n_s(t_c^-) \xrightarrow{N\to\infty} 0$, I-ii) $\sum_{s=1}^{\infty} n_s(t_c^-) \sim O(1)$, and I-iii) $\sum_{s=s*}^{\infty} sn_s(t_c^-) = r(0 < r < 1)$.

E. Numerical check of those conditions for several models showing type-II DPTs.

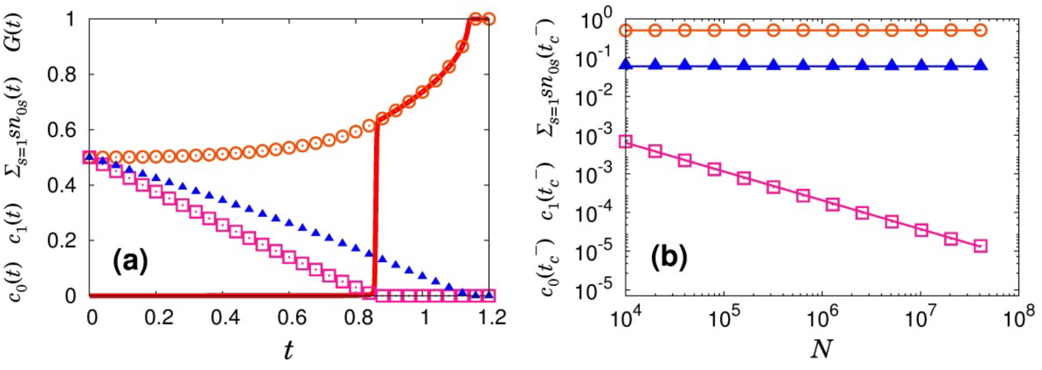

Fig. S7 E-1) (a) For the TCA model, plots of the total number of clusters of type 0 divided by N denoted as $c_0(t)=\sum_{s=1}^{\infty} n_{0s}(t)$ (□), that of 1-type denoted as $c_1(t)=\sum_{s=1}^{\infty} n_{1s}(t)$ (▲), $\sum_{s=1}^{\infty} sn_{0s}(t)$ (○), and the size of the giant cluster divided by N denoted as $G(t)$ (Solid line and curve) as a function of time t. Those quantities satisfy the necessary conditions. (b) Plots of $c_0(t_c^-)$, $c_1(t_c^-)$ and $\sum_{s=1}^{\infty} sn_{0s}(t_c^-)$ as a function of system size N. They behave as $c_0(t_c^-)\sim O(N^{\alpha-1})$ with $\alpha<1$, $c_1(t_c^-)\sim O(1)$, $\sum_{s=1}^{\infty} sn_{0s}(t_c^-)\sim O(1)$, respectively.

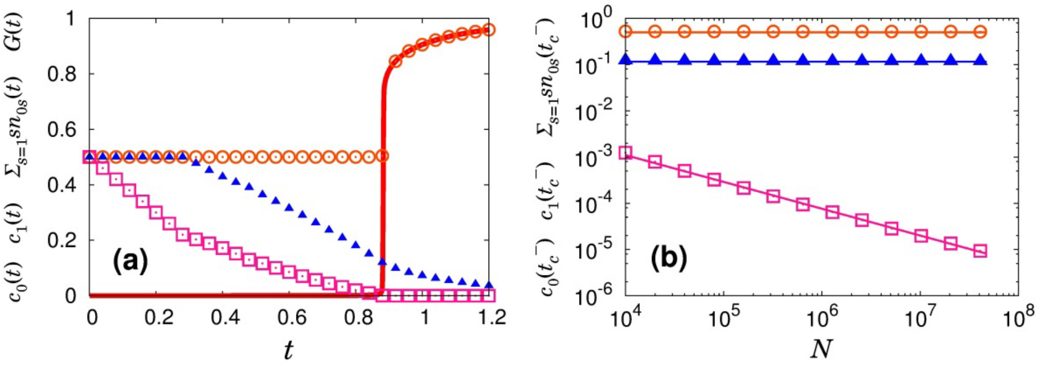

Fig. S8 E-2) (a) Similar plots for the half-restricted process model. The three quantities behave similarly as those for the TCA model. The half-restricted process model is defined as follows: At each step, we choose one node randomly from all nodes, and another node randomly from a restricted set A of nodes. The set A is defined via the formula $\sum_{s=1}^{s^*} sn_s(t) = d$, where s^* is the size of the largest cluster in the set A. That is, the number of nodes belonging to the set A is dN , where we take $d = 0.5$. The set B is defined as a set containing the rest clusters in the system. The clusters of type 0 are defined as the ones in the set B, and the clusters of type 1 are the ones in the set A. (b) Plots of $c_0(t_c^-)$, $c_1(t_c^-)$ and $\sum_{s=1}^{\infty} sn_{0s}(t_c^-)$ as a function of system size N . They behave as $c_0(t_c^-) \sim O(N^{\alpha-1})$ with $\alpha<1$, $c_1(t_c^-) \sim O(1)$, $\sum_{s=1}^{\infty} sn_{0s}(t_c^-) \sim O(1)$, respectively.

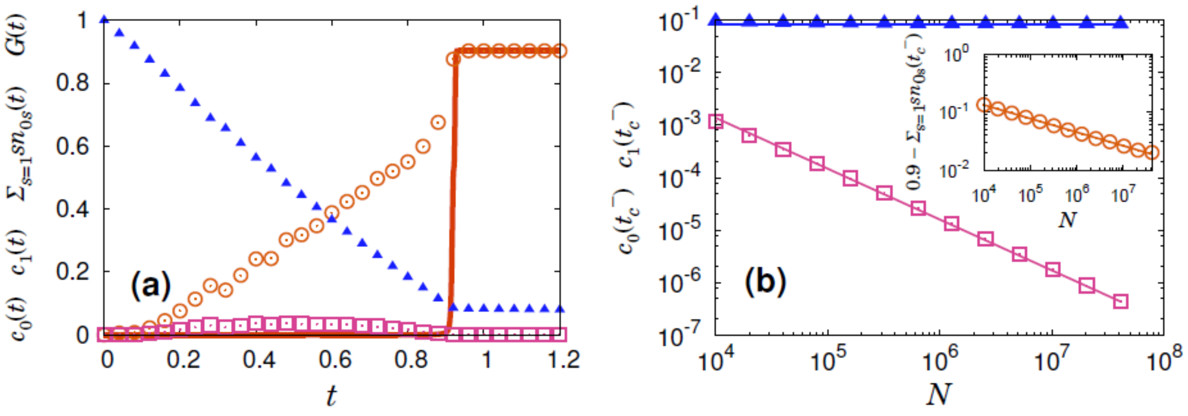

Fig. S9 E-3) (a) Similar plots for the so-called BFW model for a model parameter value $\alpha = 0.7$. In this model, the three quantities $c_0(t), c_1(t)$ and $\sum_{s=1} sn_{0s}(t)$, behave similarly as those for the half-restricted process model. The clusters of type 0 are defined as the ones that are suppressed to grow (i.e. the clusters larger than the half of the cap size given in this model). (b) Plots of $c_0(t_c^-)$, $c_1(t_c^-)$ and $\sum_{s=1}^{\infty} sn_{0s}(t_c^-)$ as a function of system size N . They behave as $c_0(t_c^-) \sim O(N^{\alpha-1})$ with $\alpha<1$, $c_1(t_c^-) \sim O(1)$ and $\sum_{s=1}^{\infty} sn_{0s}(t_c^-) \sim O(1)$, respectively.

III. Symmetry breaking dynamic rule for type-II synchronization transition

Symmetry breaking dynamic rule for synchronization transition has been already studied by J. Gómez-Gardeñes et al. (Ref. [31]). They studied a Kuramoto equation when the natural frequency of each node is identical to its degree. Thus, the distribution of natural frequency $g(\omega)$ is given as degree distribution $P(k)$. Moreover, the Kuramoto equation is applied to a network composed of two different types of networks, Erdös-Rènyi (ER) network and scale-free (SF) network with fraction of α and 1-α, respectively. One may transfer this problem to the case of the Kuramoto equation following the natural frequency distribution given as $g(\omega = k) = \alpha P_{ER}(k) + (1-\alpha)P_{SF}(k)$.

Thus the dynamic rule contains two types of natural frequencies. For this case, they observed that when $\alpha = 0.2$, at a transition point, the order parameter increases drastically up to a finite value less than unity followed by a gradual increase, reminiscent of type-II discontinuous percolation transition as we studied. This behavior can be seen in Fig.1(c) of Ref. [31]. Moreover, the authors presented the evolution of effective frequencies as a function of a control parameter in Fig.2(c). For the case $\alpha = 0.2$, a finite fraction of effective frequencies are entrained at the onset of the synchronization, however, the rest fraction of effective frequencies are not still entrained, but they begin to synchronize when the order parameter begins to increase gradually. Thus, our criterion for the type-II discontinuous percolation transition seems to be fulfilled heuristically in type-II synchronization transition.
